# Protein Hydrolysates as Promoters of Non-Haem Iron Absorption

**DOI:** 10.3390/nu9060609

**Published:** 2017-06-15

**Authors:** Yanan Li, Han Jiang, Guangrong Huang

**Affiliations:** 1 College of Life Sciences, China Jiliang University, Hangzhou 310018, China; dreamforlyn@gmail.com (Y.L.); jianghan@cjlu.edu.cn (H.J.); 2 Key Lab of Marine Food Quality and Hazard Controlling Technology of Zhejiang Province, Hangzhou 310018, China; 3 National and Local United Engineering Lab of Quality Controlling Technology and Instrument for Marine Food, Hangzhou 310018, China

**Keywords:** amino acid, di-peptide, tri-peptide, polypeptide, iron chelate, food supplement, bioactive peptides

## Abstract

Iron (Fe) is an essential micronutrient for human growth and health. Organic iron is an excellent iron supplement due to its bioavailability. Both amino acids and peptides improve iron bioavailability and absorption and are therefore valuable components of iron supplements. This review focuses on protein hydrolysates as potential promoters of iron absorption. The ability of protein hydrolysates to chelate iron is thought to be a key attribute for the promotion of iron absorption. Iron-chelatable protein hydrolysates are categorized by their absorption forms: amino acids, di- and tri-peptides and polypeptides. Their structural characteristics, including their size and amino acid sequence, as well as the presence of special amino acids, influence their iron chelation abilities and bioavailabilities. Protein hydrolysates promote iron absorption by keeping iron soluble, reducing ferric iron to ferrous iron, and promoting transport across cell membranes into the gut. We also discuss the use and relative merits of protein hydrolysates as iron supplements.

## 1. Introduction

Iron (Fe) is an essential micronutrient for human growth and health. Iron affects the health of children, the development of teenagers and the immune system of adults. Iron also plays a role in many cellular metabolic activities, such as carrying oxygen in haemoglobin and myoglobin and transporting electrons in the various cytochrome systems, as well as in ferredoxin for respiration. Anaemia and iron deficiency reduce an individual’s well-being, cause fatigue and lethargy, and impair physical capacity and work performance. Maternal anaemia is associated with mortality and morbidity in the mother and baby, including increased risks of miscarriages, stillbirths, prematurity and low birth weight [1]. However, iron deficiency and iron deficiency anaemia (IDA) are classified as the most prevalent nutritional disorders in the world by the WHO; they affect more than 3.5 billion people in the developing world [2]. Almost half of children are anaemic, most of whom live in undeveloped countries [3]. More than 20% of women experience iron deficiency during their reproductive lives [4].

Inadequate iron intake and absorption are the main causes of iron deficiency, which leads to IDA. In these cases, iron supplements or iron fortifiers are needed to overcome the iron deficiency. Commercially available oral iron supplements include ferrous sulfate, ferrous gluconate, ferrous fumarate, iron dextran, and other iron-containing compounds.

Organic iron is thought to have a better bioavailability and have fewer side effects than inorganic iron salts. For example, for a diet containing only 6% of its total iron as haem, 30% of the iron absorbed was acquired from haem to the exclusion of other dietary iron sources [5]. Alternatively, iron can also chelate with a sugar as an iron supplement, such as iron sucrose (Venofer^®^), iron polysaccharide (Niferex^®^), iron dextran and iron carboxymaltose (Ferinject^®^). These iron-sugar complexes have special advantages, such as their minimal side effects. For example, iron-polymaltose complexes have a bioavailability similar to that of ferrous salts, and are preferable in terms of their balance between efficacy and toxicity [6]. The milks fortified by iron sulfate stabilized with maltodextrin covers toddlers’ requirements of iron [7].

Proteins and their hydrolysates are important organic substances, and mineral chelating peptides have the ability to enhance the bioavailability of minerals [8]. Some protein hydrolysates have been used in the iron fortification of food for humans and livestock. Thus, the study of protein hydrolysates as promoters of iron absorption is important.

## 2. Iron-Chelatable Protein Hydrolysates

Protein hydrolysates are protein fragments produced via hydrolysation and include amino acids and peptides of different sizes. Some hydrolysates can be synthesised or produced through bioengineering depending on their structure. Proteins are hydrolysed by enzymes or chemicals to improve their nutritional value or to search for bioactive peptides. Huge proteins, such as collagen, are hydrolysed by enzymes to improve their bioavailability. Many types of fisheries by-products are hydrolysed to change non-edible proteins into edible peptides. Proteins are also hydrolysed by acids or bases to produce amino acids. Protein hydrolysates have various structures and can be produced in large amounts. They serve as important nutrients and food ingredients, as well as playing other roles, and they are an important resource for us to further develop and utilise.

The positive effects of protein hydrolysates on the absorption of minerals, such as iron or other metals, have been reported in vivo and in vitro. The chelation ability of protein hydrolysates is thought to be a key factor in the promotion of iron absorption. Through metal chelation, peptides or amino acids increase the solubility and bioavailability of metals. Therefore, iron-chelatable protein hydrolysates are potential promoters of iron absorption. We will focus on peptides and amino acids, which have the ability to chelate iron.

Proteins are digested into oligopeptides and amino acids in the digestive tract. Amino acids, di-peptides, tri-peptides and polypeptides all have different absorption routes, except for the paracellular route. We therefore classify iron-chelatable protein hydrolysates into the three classes listed in Table 1, which are the absorbable forms of protein hydrolytic products. Some of the reported iron-chelatable protein hydrolysates are listed in Table 1.

### 2.1. Iron-Chelatable Amino Acids

Amino acids, the building blocks of proteins, are basic nutrients for all forms of life. They are an important form of protein hydrolysate that are absorbed. Amino acids can be absorbed and transported by multiple transporters, which have been identified and classified in the past several decades. As they are important nutrients that can be absorbed by cells directly, amino acids with iron-absorption promoting abilities will be promising candidates for iron supplements.

Many amino acids have been studied to determine their interactions with iron, such as one study of the equilibrium of L-glutamic acid and L-serine with iron(III) in solution [15]. Fe(His)_2_, Fe(Gly)_2_, and Fe(Arg)_2_ have been studied as iron complexes [9]. In addition, the enhanced effect of histidine, cysteine, and lysine on iron absorption is thought to be based on the tridentate chelates then form with iron [11]. Some of the reported iron-chelatable amino acids are listed in Table 1, such as methionine, glutamine, and aspartic acid. Of all the amino acids-iron complexes, iron-glycine is the most reported. The iron-glycine chelate has been proven to have a positive effect on iron absorption in piglets, rats, broilers and humans [13]. 

### 2.2. Iron-Chelatable Di-Peptides and Tri-Peptides

Di-peptides and tri-peptides, similar to free amino acids, are also important nutrients and can be transported intact into epithelial cells by the special transporter PEPT1 [57]. Furthermore, small peptides (di-peptides and tri-peptides) are the major forms of protein that are absorbed by cells. The concentration of oligopeptides (di-, tri- and tetra-peptides) in the intestinal lumen is three to four times that of free amino acids [58]. In addition, the transfer efficiency of PEPT1 is higher than that of the amino acid transporters for some substrates [59]. Thus, di- and tri-peptides are more important candidates for iron supplements than amino acids.

Many types of di- and tri-peptides have been reported to chelate iron, and some of them have been isolated from protein hydrolysates. For example, Ser-Met, Leu-Ala-Asn and Asn-Cys-Ser, which were isolated from sesame protein hydrolysates, can chelate iron to a similar degree as reduced glutathione [19]. Two iron-chelatable tri-peptides, Ser-Cys-His and His-Tyr-Asp were isolated from hydrolysates of Alaskan pollock skin collagen [23] and hairtail protein [21], respectively. Some synthesized small peptides that have iron chelation abilities have also been reported. For example, aspartame (*N*-l-α-aspartyl-l-phenylalanine methyl ester) can interact with ferrous iron, and aspartame-ferrous iron complexes have been synthesized [10]. Fe-carbamyl glycine [20] and Arg-Glu-Glu-iron [18] have also been synthesized and studied as iron supplements.

### 2.3. Iron-Chelatable Polypeptides and Proteins

Polypeptides and proteins, which are polymers of amino acids, are also important for human and animal nutrition. Polypeptides can be absorbed intact except for some forms of small peptides and amino acids, and they can be absorbed via sodium-coupled oligopeptide transporters (SOPT 1 and SOPT2), paracellular passive transport, transcellular passive diffusion and transcytosis [60]. Proteins, similar to polypeptides, can also be absorbed by the paracellular and transcellular pathways. Insulin, for example, can be absorbed by the small intestine at its apical side via endocytosis [61].

Iron-chelatable polypeptides are also important iron supplement candidates; therefore, we will further discuss polypeptides and proteins. The variations are countless for peptides consisting of four or more amino acids, and therefore, iron-chelatable polypeptides are also innumerable. Generally, iron-chelatable polypeptides can be classified into several groups: proteins, protein hydrolysates and other polypeptides.

#### 2.3.1. Iron-Chelatable Proteins

Approximately 30% of proteins and enzymes contain metal or metalloid ions in their structures. Most of these proteins contain an iron or iron-like metal ion because they contain an amino acid motif that can chelate iron. Many proteins, such as thiolated human-like collagen, can chelate iron, as can the iron metabolism-related proteins [55]. In iron-enriched baker’s yeast and soybeans, iron also binds proteins [53,62]. Some complexes, such as iron-bound whey proteins, have good stability under different processing conditions [56].

#### 2.3.2. Iron-Chelatable Protein Fragments

The iron-chelating subunits of proteins in food can be released by cooking, digestion and hydrolyzation. These released protein hydrolysates or their fragments are potential iron supplements due to their iron chelation abilities. Proteins that are inexpensive and easy to obtain are major sources for the production of metal-chelatable peptides or other bioactive peptides. Proteins from cereals, aquatic products, milk, and other sources can be hydrolysed by many different types of enzymes to identify iron-chelatable peptides. 

For example, a polypeptide from a barley protein, Ser-Val-Asn-Val-Pro-Leu-Tyr, spontaneously forms a complex with an iron ion at physiological pH [25]. Several iron-chelatable peptides were identified from soybean proteins that were hydrolysed by pepsin, trypsin, protease, deamidase and other enzymes [49,51]. Hydrolysates of shrimp, fish and seaweed also have the ability to chelate iron, and several iron-chelatable peptides have been isolated from these sources [32,43,45,46]. 

In particular, phosphopeptides from casein [28], egg white [63] and other similar sources make up one category of peptides that have the ability to chelate iron. Peptides derived from collagen also have the ability to chelate iron. This category includes peptides from the skin of Alaskan pollock [23,24] and cod [32] and the scales of *Latescalcarifer*, *Mugilcephalus*, *Chanoschanos*, and *Oreochromis* spp. [64].

#### 2.3.3. Other Iron-Chelatable Peptides

In addition to the two categories discussed above, many natural and synthetic peptides have also been studied. Cell-penetrating peptides that can efficiently translocate through the plasma membrane are able to deliver cargos across the membrane both in vitro and in vivo [65]. These cargos range from small to large molecules and can include medicines and proteins. An iron ion can also be cargo. The tri-peptide Arg-Glu-Glu [18] was designed using the rules governing cell-penetrating peptides; the Arg residue improves the penetrability of the peptide and has been proven to promote iron absorption in the form of a chelate. Peptides produced by microorganisms have also been reported to contain iron or to have the ability to chelate iron. Four kinds of ferrocins, which are iron-containing peptides, have been found in one species of gram-negative bacterium [66]. Probiotic bacteria grown in culture media with different nitrogen sources have been shown to produce iron-binding peptides [67]. Iron-binding peptides from *Aspergillus versicolour* [68], *Aspergillus oryzae* [33] and *Lactococcus lactis* [38] are all thought to be promising bioactive peptides that are able to promote iron absorption.

### 2.4. Structural Characteristics of Iron-Chelatable Protein Hydrolysates

Many protein hydrolysates can chelate iron. However, the protein hydrolysate constituents that are responsible for chelating iron are identified randomly, which is inefficient. Nonetheless, identifying the active components of these protein hydrolysates is necessary to find or synthesize a peptide that has the ability to chelate iron. The chelates of protein hydrolysates and iron ions are complicated because both peptides and amino acids are amphoteric molecules. Protein hydrolysates contain cationic, anionic and zwitterionic forms of peptides at different pH values, and iron ions have different valence states (Fe^2+^ and Fe^3+^). However, all protein hydrolysates have a similar chemical nature: terminal amino and carboxyl groups with various side-chains. Iron ions have limited differences in their extra-nuclear electron configurations. Therefore, there are rules that can be followed to identify or design peptides. Iron ions acting as a Lewis acid can react with oxygen-rich and nitrogen-rich groups, which are Lewis bases. Fe^2+^ can be classified as a borderline Lewis metal ion, and Fe^3+^ belongs to the hard group of Lewis metal ions. According to this rule, Fe^3+^ prefers oxygen-rich groups, such as the carboxyl groups and phosphate groups (which are hard Lewis bases), and Fe^2+^ has a preference for nitrogen-containing groups [69].

#### 2.4.1. Structural Characteristics of Iron-Chelatable Amino Acids

Every natural amino acid has two effective donor groups (amino and carboxyl) and is capable of forming a stable, five-membered chelate ring with a metal atom [70]. In addition to these two groups, the side-chain of an amino acid (R group), which defines each amino acid, also plays an important role in determining the chelate that is formed. In general, the R group affects the complex by changing the chemical environment of the amino and carboxyl groups. Furthermore, the electron rich R groups of some amino acids, such as the imidazole group of histidine and the sulfhydryl group of cysteine, can also participate in chelation. Additionally, Glu and Asp prefer to form chelates with Fe^3+^ at their oxygen-rich R groups; however, Arg and Asn prefer to form chelates with Fe^2+^ at their oxygen-rich R groups.

#### 2.4.2. Structural Characteristics of Iron-Chelatable Peptides

Peptides have many variants, and their iron chelates are more complicated. There are several factors affecting the stability of these chelates.

##### Special Amino Acids

Certain special amino acids have strong iron chelation abilities, and peptides containing these amino acids have higher iron chelation abilities than other peptides. The activity of these iron chelates is related to these special amino acids, and those special amino acids can also determine whether they prefer Fe^3+^ or Fe^2+^. Peptides containing Glu and Asp have higher affinities for Fe^3+^, and peptides containing Arg and Asn prefer to form chelates with Fe^2+^.

His has a strong metal chelating ability due to its imidazole group. Peptides that are rich in His have higher iron-chelating activities than other peptides in the hydrolysates of some proteins [31,43,71]. Serine is also an important amino acid that affects the stability of iron-containing peptides, likely due to its hydroxyl group [23]. Peptides containing Ser have higher iron and zinc chelating abilities [19,43]. Similar to serine, cysteine also contributes to iron-chelating activity of a peptide due to its sulfhydryl group. In peptides derived from meat protein, Cys has been recognized as an important amino acid that promotes iron absorption through its chelating activity [58,72]. Peptides containing Cys also show higher activities for iron and zinc chelation than other peptides [19]. Phosphorylated amino acids, especially phosphoserine, make up another category of important amino acids since they can create suitable chelating sites for positively charged iron ions [23]. Caseinophosphopeptides (CPPs) derived from milk proteins contain a high proportion of phosphoserines [73] and can stably chelate iron. Asp and Glu have also been reported to contribute to the chelating ability of peptides due to their carboxyl groups.

##### Size of the Peptides

Only a single amino or carboxyl group is available at each terminus of a peptide, while other amino and carboxyl groups exist within the peptide bonds that connect the amino acids of the peptide. If the peptides are smaller in size, the proportion of amino and carboxyl groups (the oxygen of the C-terminus and the nitrogen of the N-terminus) will be higher, and the iron chelation activity may be higher as well, and it has been shown that peptides with lower molecular weights have higher iron-chelating activities in protein hydrolysates of *P. columbina* [43]. The iron-binding capacity of sea cucumber (*Stichopus japonicus*) ovum hydrolysates increased significantly, from 55.7% to 92.1%, as their molecular weight decreased and as the proportion of fractions larger than 1000 Da decreased markedly from 58.5% to 36.4% [74]. Conversely, for large peptides, the terminal groups represent a very small proportion of the total peptide mass and can result in these peptides having lower chelation abilities. However, some large peptides contain special amino acids, and they actually have stronger iron chelation abilities due to the greater amount of dentate areas. The proper size of a peptide is determined by both the content of special amino acids and the other aforementioned factors. Thus, peptides with a proper size that offer higher ratios of dentate areas will have higher iron chelation abilities. 

##### Sequences of the Peptides

When peptides, as well as those with more dentate areas, are composed of certain special amino acids, these peptides have low iron-chelating activities, which is inconsistent with the rules stated above. In this situation, the peptides may not be in an appropriate sequence. Chemical substances prefer to form a thermodynamically stable five- or six-member ring. Furthermore, some R groups are large or have strong charges that can affect the stability of the chelate. Thus, the side-chains and their positions or sequences will also affect the chelation ability of a peptide. 

In summary, almost all amino acids can chelate iron ions; however, the stabilities of the chelation complexes vary due to the R groups of the amino acids and their chemical environments. The chelates of peptides with iron can be affected by the content of special amino acids and the size and sequence of the peptide. The equilibrium constants of the complexes are very different for different substrates and environments. Only some peptides have high equilibrium constants and can stably chelate with iron. In the future, a model that predicts the equilibrium constant of an iron complex could be designed computationally. Then, with the help of bioinformatic methods, we could choose and produce iron-chelatable peptides from food proteins or synthesize one purposefully. At such a time, our ability to identify and use iron-chelatable protein hydrolysates will progress more efficiently.

## 3. Mechanisms of Promoting Non-Haem Iron Absorption

### 3.1. Iron Absorption

**Figure 1:**
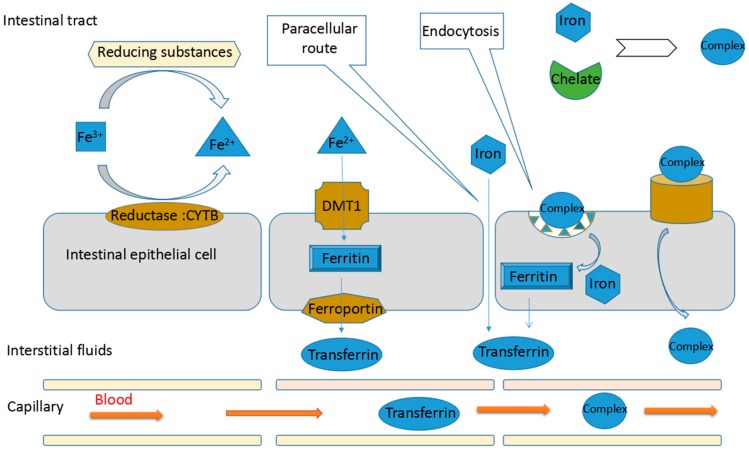
Mechanisms of iron absorption. Iron is absorbed both in ion and complexed forms, as well as via the paracellular route. For iron ions, Fe^3+^ must be reduced to Fe^2+^ by reductases, such as cytochrome *b*, and then be absorbed by the divalent metal transporter 1. The iron is stored in ferritin, transported out of cells by ferroportin and distributed by transferrin. In addition, iron (both Fe^2+^ and Fe^3+^) can chelate other molecules and be absorbed in the form of complexes via endocytosis and importers, after which their fate includes transformation to iron ions and transfer in their complexed form.

In mammalian systems, iron absorption differs significantly with various host- and diet-related variables, including the life-stage and iron status of the organism, as well as the enhancers and inhibitors of iron absorption present in the consumed food. However, dietary iron can be absorbed in the ion and molecular form irrespective of the paracellular route. For the iron ion, Fe^3+^ must be reduced to Fe^2+^ by a reducing substance, such as cytochrome *b* or another reductase on the brush border membrane, or by reductants in our food or gastrointestinal secretions. Then, Fe^2+^ is internalized by enterocytes via the apical transporter divalent metal transporter 1 (DMT1). The iron is stored as ferritin inside the enterocytes and can be transported to the interstitial fluids by the basolateral transporter ferroportin when necessary. The iron is then distributed throughout the body in the form of transferrin-bound iron via the circulatory system [75]. In addition, these iron ions can chelate other molecules and be absorbed in a molecularly bound form, such as polysaccharide-iron complexes [76]. In general, the absorption of molecular iron occurs through either endocytosis or importers. For example, haem-iron is absorbed in the form of haem, and its absorption occurs mainly via receptor-mediated endocytosis that is partially mediated by the proton coupled folate transporter (PCFT) or other unidentified low-affinity haem importers [77]. In intestinal epithelial cells, some internalized iron chelates are catabolized to liberate Fe^2+^, similar to haem, which then follows the fate of dietary Fe^2+^. Of course, some iron chelates may be transferred in their unmodified molecular form, just as some haem can be transported intact to the plasma. The mechanisms of iron absorption are shown in Figure 1.

### 3.2. Mechanisms of Protein Hydrolysates Promoting Non-Haem Iron Absorption

Currently, three theories exist regarding how protein hydrolysates promote iron absorption in mammalian systems. Protein hydrolysates are thought to maintain the solubility of iron, to reduce ferric ion to ferrous ion and keep iron at a low valence state, and/or to promote iron uptake through intestinal cell membranes. The first two theories involve increasing the concentration of soluble iron to promote the entrance of iron into enterocytes through the DMT1 receptor, whereas the latter theory suggests that protein hydrolysates mediate the absorption of bound iron through a peptide or amino acid transporter localized in the brush border membranes.

#### 3.2.1. Maintaining the Solubility of Iron

All nutrients must be absorbed in solution. However, our diet generally contains iron absorption inhibitors, such as phytic acid, tannins, oxalate and polyphenols, which can chelate iron ions and decrease their solubility [78]. In addition, ferric iron becomes insoluble at pH values greater than 3.0, and the pH in the intestinal lumen is basic.

Peptides and amino acids can chelate iron, and their complexes protect iron ions from these inhibitors and the conditions in the fluid of the small intestine, keeping the iron ions in solution. For example, Cys and reduced cysteinylglycine can significantly increase the solubility of iron in a solution containing insoluble iron [58]. Additionally, the hydrolysates of red seaweed (*P. columbina*) protein can maintain iron in a soluble and bioaccessible form after gastrointestinal digestion [43]. CPPs derived from milk proteins with a special sequence of three phosphoseryl residues followed by two glutamic acid residues, Ser(P)-Ser(P)-Ser(P)-Glu-Glu, act as mineral absorption enhancing peptides [8,79,80]. The binding of iron to CPPs increases iron solubility in the alkaline intestinal environment and influence how accessible iron is to apical membranes [81].

#### 3.2.2. Reducing Ferric Ion to Ferrous Ion

As discussed in Section 3.1, most iron ions must be reduced to ferrous ions before being transported by DMT1. Some reductive or antioxidant peptides and amino acids promote iron absorption by reducing ferric iron to ferrous iron, just like ascorbic acid. In addition, Cys and reduced cysteinylglycine enhance ferric iron absorption in Caco-2 cells, but they have no positive effect on ferrous iron [58], suggesting that they may promote iron absorption by reducing ferric iron.

#### 3.2.3. Promoting the Passage of Iron through Intestinal Cell Membranes

Thus far, we have focused on discussing ways to increase the concentration of ferrous iron that arrives at intestinal cell membranes, and we will now consider approaches for promoting the uptake of iron through intestinal cell membranes. Protein hydrolysates have the potential to be excellent iron absorption promoters. Peptides and amino acids have special transporters or pathways in the brush border membranes, and they may carry iron ions when they are absorbed. This absorption of iron is not related to DMT1, but does increase overall iron absorption. For example, Fe-Gly has a special transit system that is different from the absorption system of FeSO_4_ [5,82,83]. The tri-peptide iron complex Arg-Glu-Glu-Fe, an effective iron supplement for IDA rats, was designed as a cell-penetrating peptide [18]. Some CPP-iron complexes seem to be absorbed via endocytosis in vivo [84].

In conclusion, protein hydrolysates improve iron absorptionin three ways: maintaining the solubility of iron, facilitating the conversion of ferric iron to ferrous iron and promoting the absorption of iron through the intestinal cell membranes. In other words, peptides and amino acids can maintain the solubility of iron through their chelation and reducing abilities. Some complexes can also be absorbed in the form of molecules via PET1, endocytosis, and other modes. 

## 4. Usages, Advantages and Challenges of Protein Hydrolysates as Non-Haem Iron Promoters

### 4.1. Usagesof Protein Hydrolysates as Non-Haem Iron Promoters

Protein hydrolysates, especially those derived from food protein, are safe when used as iron supplements. Iron chelates of protein hydrolysates have attracted a great amount of attention as a new type of iron supplement [18]. Amino acids [12], small peptides [85] and polypeptides [36] have been confirmed to be able to improve iron bioavailability or absorption. They also have the potential to be used in the food and feed industries, and many of them are already being used. Some of the peptides and amino acids that have been used as iron supplements are listed in Table 2.

#### 4.1.1. Amino Acidsas Non-Haem Iron Promoters

Many types of amino acids have been reported to promote iron absorption at the cellular and organismal level, including in humans. Both the full spectrum of amino acids and single amino acids have been shown to promote iron absorption in research and commercial usage. 

Aspartic acid, glutamic acid and histidine enhance the uptake and transport of iron by Caco-2 cells [91]. Histidine, cysteine, and lysine enhance in vivo iron uptake in segments of rat duodenum [11]. Iron-amino acid chelates provided faster rates of improvement in haemoglobin levels and were better tolerated by the patients than ferrous-fumarate in a randomized controlled study [92]. Multi-amino acid-iron chelates have been used in premenopausal women and preschool children and have shown positive effects in combating iron deficiency and reducing the number of adverse effects [86,93]. 

Of all the amino acids, glycine is the most widely used iron chelation ligand. The iron in Fe-Gly can be more easily absorbed than FeSO_4_ in Caco-2 cells [82]. An addition of Fe-Gly to feed mixtures for broilers contributed to significant changes in the level of biochemical and haematological indicators in their blood [13,94]. Ironbis-glycine chelatesare a suitable compound for food fortification as they prevent the inhibitory effect of phytates [95]. Ferrous bis-glycine chelates can be used for iron fortification in milk as they improve haemoglobin and ferritin serum levels and do not alter milk’s organoleptic properties [96]. Furthermore, ferrous bis-glycine can be used in high-phytate foods [12].

#### 4.1.2. Di-Peptides and Tri-Peptidesas Non-Haem Iron Promoters

Di- and tri-peptides can be absorbed quickly. Although there are few reports about the use of di-peptide and tri-peptide iron chelates as iron supplements, it is still a promising direction for us to study. Di-peptides, anserine and carnosine enhanced the uptake and transport of iron by Caco-2 cells [91]. Glutathione also possess iron absorption-enhancing ability [97]. The Arg-Glu-Glu-Fe complex is an effective iron source for IDA rats [18]. Fe-carbamyl glycine is used as an iron fortifier in feed [88]. 

#### 4.1.3. Polypeptidesas Non-Haem Iron Promoters

Complexes synthesized with low-molecular-mass peptides (<5 kDa) and FeCl_2_ increased iron uptake by approximately 70% compared with uptake of FeSO_4_ in a Caco-2 cell model [52]. Caco-2 cellular uptake increased 4-fold for the Fe^2+^-(Ser-Val-Asn-Val-Pro-Leu-Tyr), a barley-derived peptide complex, after pepsin-pancreatin digestion compared to the uptake of iron sulfate salt [25]. Iron-binding peptides derived from sericin have been shown to improve iron bioavailability and hasten the alleviation of iron deficiency in experimental rats [44]. Ferrichrysin (an iron-chelated cyclic peptide) exhibited the same beneficial effect in improving IDA as ferric citrate, being significantly greater than the effect of haem iron in anaemic Sprague–Dawley (SD) rats [33]. Cysteine-containing peptides derived from meat have apromoting effect on the absorption of non-haem iron in human [72]. Deferrichrysin can be used in food as a food supplement, to prevent colour change and to create iron-fortified foods [89]. A component of traditional Chinese medicine, A ’Jiao (*Collacoriiasini*, donkey-hide gelatine), has been used to enrich the blood for thousands of years in China [90]. Egg white protein is useful for recovery of IDA in SD rats [98]. Soybean sprouts fortified with iron (ferritin) are a good iron supplement with no side effects [53]. Iron-enriched baker’s yeast, which contains iron bound to proteins in the yeast cells, is more efficient than inorganic iron in treating anaemic rats [62].

The CPPs derived from casein are important sources of mineral-chelating peptides. The addition of 5–10 g CPPs/100 g soya flour enhanced the level of bioaccessible iron in native and iron-fortified flour to a significant extent [30], and the addition of CPPs in a milk system improved iron binding abilities [99]. Fe uptake compared to that of FeSO_4_ was significantly increased in tissues (liver, spleen and sacrum) when Fe-β-CN(1–25)4P or FeSO_4_ was administered once to 10 young females (20–30 years) [100].

Collagen, a major protein constituent of skin, cartilage, and tendons, is also an important source of mineral-chelating peptides. Collagen peptides derived from by-products of *Gadus chalcogrammus*, *Lates calcarifer*, *Mugil cephalus*, *Chanos chanos*, and *Oreochromis* spp. are reported to have iron-chelating ability [24,64]. Collagen peptides derived from deer sinew and fish scales have calcium (calcium is similar to iron) absorption-promoting effects [101,102]. GPAGPHGPPG has been shown to have significant promotional effects on iron transport in Caco-2 cell monolayers [103].

Besides casein and collagen, other proteins, such as soy protein, fish protein, are also hydrolyzed and the resulting hydrolysates have been studied as iron chelation or iron absorption-promoting peptides.

In conclusion, all of these protein hydrolysates have representative materials that are used in iron supplements, with their structure deciding their properties, but it is hard to compare their promoting effect. However, based on the number of variants, peptides have more advantages for use in iron supplements. Furthermore, di-peptides and tri-peptides have faster absorption rates than amino acids and are more easily absorbed than polypeptides. Therefore, the small peptides, as well as some larger peptides that can be digested down to small peptides, may be more suitable for use in developing new iron supplements.

### 4.2. Advantagesof Protein Hydrolysates as Non-Haem Iron Promoters

#### 4.2.1. Dual-Purpose Nutrients

Protein hydrolysate iron complexes provide iron for humans and other animals. At the same time, ligands of iron, amino acids and peptides are also important nutrients. A major nutrient, proteins and their hydrolysates can be used to synthesize the basic materials needed by our bodies and can be metabolized to provide energy when they are absorbed. Moreover, peptides and amino acids are safer than other chemical substances in the conditions that they are absorbed and utilized.

#### 4.2.2. Reducing the Side Effects of Iron Ions

Peptides and amino acids with iron-chelating activities can also reduce the production of reactive oxygen species (ROS). ROS are generated in Fenton reactions where iron or other metal ions are involved [31]. ROS are related to the off-flavour of foods [104], as well as a variety of pathologic situations [105]. The reason for this is that ROS promote destructive free-radical reactions in foods or in our bodies. Thus, chelatable protein hydrolysates maintain the quality of foods and reduce the risk of disease while increasing the bioavailability of iron.

Excess iron can be extremely toxic to animals because it catalyses the generation of ROS [106]. Under iron-overload conditions, iron is deposited in organs, such as the liver, heart and pancreas, and damage can then be caused by the production of free radicals. However, protein hydrolysates can reduce the damage caused by iron overload. The compound ferrous gluconate, stabilized with glycine, has a higher liver iron content than ferrous sulfate, and its LD_50_ (median lethal dose) is six times higher than that of ferrous sulfate in SD rats [107]. Glutamylcysteine has been shown to protect the liver against iron overload-induced injury in an iron-overload rat model due to its antioxidant properties and chelation ability [108]. In addition, yeast that is enriched with iron is a less toxic iron source than other iron sources [109].

#### 4.2.3. Bioactivity

Protein hydrolysates and their iron chelates may have some special functions for people and animals, such as acting as an antioxidant and improving immune system activity. 

Some peptides or amino acids, such as glutathione, have antioxidant functions and can reduce the production of ROS in vitro and in vivo. Fe-Gly improves the antioxidant status of broiler chickens [110] and protects hypobaric hypoxia-induced tissue injury [111]. S-Allyl cysteine, a sulphur containing amino acid derived from garlic, has a protective effect against alterations to iron metabolism induced by oxidative stress in diabetic rats [112]. Histidine di-peptides, carnosine and *N*-acetyl-carnosine significantly reduce ferritin aggregation and protect against salsolinol-mediated ferritin modification, which is the consequence of free radical scavenging activity [113]. For example, carnosine has antioxidant properties, and it has the ability to react with ROS, reactive nitrogen species and harmful aldehydes [114].

Some protein hydrolysates chelated with iron can improve immune system function. An example is Fe-Gly, which stimulates cellular defence mechanisms by increasing the percentage of Th1 cells and by enhancing the production of cytotoxic CD8+ T cells and IL-2 [115]. The complexing of di-peptide isoleucyl-tryptophan with Fe^2+^ has been studied as an immunomodulatory formulation [22], and hairtail protein hydrolysate-Fe^2+^ complexes increase the growth and non-specific immunity of crayfish [35].

Other functions may also be possessed by protein hydrolysate-iron chelates. For example, ferrous-amino acid chelates can effectively lower blood glucose and improve insulin sensitivity [116], and the collagen peptide-iron complexes may function in promoting skin and bone repair, which is a property possessed by collagen peptides.

In conclusion, complexes of peptides and amino acids with iron are promising iron supplements for three reasons. First, peptides and amino acids are important for nutrition; second, they can decrease the side effects of iron by decreasing the ROS produced by iron ions, as well as the damage caused by iron overload; and third, peptides, amino acids and their iron complexes have special functions and can be used as bioactive ingredients in foods.

### 4.3. Challenges of Protein Hydrolysates as Non-Haem Iron Promoters

Although many peptides and amino acids have been confirmed to promote iron absorption, some problems still need to be overcome before these iron complexes can be commercialized.

The crucial problem is the stability of the iron complexes. Iron supplements undergo many changes as they are surrounded by many materials during long periods of storage and processing. An iron casein succinylated liquid oral preparation has an unpalatable taste after a long period of storage [117]. Similarly, peptides and amino acids also face the same issues. In addition, the released iron ions undesirably change the colour of water and milk to dark grey. Gastrointestinal stability is also one of the problems that must be overcome. The changes to the chelates that occur during passage through the gastrointestinal tract are still not clear, and we do not know what form of iron arrives at the small intestinal epithelium. A significant proportion of iron in iron bis-glycine chelates is released in the stomach at a low pH [118]. In other words, data concerning the compatibility of peptides, amino acids and hydrolysates with different food matrices, as well as data on their stability during gastrointestinal passage and long-term storage, are needed.

Furthermore, the mechanisms by which protein hydrolysates promote iron absorption remain unclear, and more details are needed. Are there any protein hydrolysate iron complexes that can be absorbed by small intestinal cells via PEPT? Perhaps other transporters and their proportional contributions to total iron absorption should be studied. There are too many iron-chelatable peptides for us to choose from, and new peptides are still being reported. However, we do not know which class of protein hydrolysates or which kind of peptides (amino acids) have better iron promoting effects. It is hard to select the perfect peptide or amino acid for an iron supplement if we do not know its precise mechanism. Furthermore, amino acids and peptides are zwitterions, and they have different dissociated states at different pH values. In addition, they can form one or more rings with iron ions under different conditions. In these cases, their iron complexes will exist in many variations with different equilibrium constants, which is also an issue for their usage. Although most protein hydrolysate iron complexes have been shown to have iron supplementation effects on animals or cells, their use for nutritional or medical purposes are just theoretical and have only been hypothesized. Therefore, there are still additional factors to determine for their use in humans, which need to be tested.

In addition, safety is also a problem. Although protein hydrolysates are relatively safe, some protein hydrolysates may still be allergens, which may cause immunoreactions. Furthermore, the sources of peptides also affect their safety. Non-food proteins and contaminated food proteins, such as venom proteins or proteins from animals with prions, are not safe sources of protein hydrolysates. 

## 5. Conclusions

Protein hydrolysates are promising iron supplements in the form of iron chelates. Iron deficiency is a global issue. The work to find effective and safe iron supplements is never-ending. Much of the literature focuses on protein hydrolysates, which have a promoting effect on iron absorption. Iron chelation ability is thought to be a key factor for the chelating effect of protein hydrolysates. We reviewed the reported iron-chelatable protein hydrolysates and described their structural characteristics. These amino acids and peptides show us that there are abundant sources of iron-chelatable protein hydrolysates. Their relevant characteristics, which include special amino acid compositions, as well as size- and sequence-dependent peptide properties, can guide us to find or synthesize potential iron-chelatable protein hydrolysates.

Protein hydrolysates promote iron absorption in three ways: maintaining the solubility of iron, reducing ferric ions to ferrous ions to keep iron at a low valence state, and promoting the absorption of iron through intestinal cell membranes. Maintaining the solubility of iron, which is related tochelation ability, is of great concern. Reducing ferric ion to ferrous ion with protein hydrolysates or other substrates is also important. However, the method by which iron is carried through the intestinal cell membrane has not been sufficiently studied, and more research should focus on this problem.

Protein hydrolysates can be used in iron supplements as safe food ingredients. Some amino acids, peptides and proteins have been used as iron supplements. Furthermore, protein hydrolysates have some excellent characteristics. First, they can be used as nutrients while promoting iron absorption. Second, their chelation ability can protect individuals from the side effects of iron ions and reduce the damage caused by iron overload. Lastly, they have bioactivities alone and when complexed. Although some protein hydrolysates have been used as iron supplements, many problems still need to be overcome. Their compatibility with different food matrices must be studied systematically. Both their gastrointestinal and long-term storage stabilities also require further investigation. The details of how the complexes are absorbed are still not clear, and we have no rules by which to choose the best peptide or amino acid iron supplement among all of the candidates.

We predict that more and more organic iron supplements will be used and that peptide-iron complexes and amino acids-iron complexes will become popular in the market of iron fortifiers. Similarly, the bioavailability of other minerals, such as zinc, calcium and copper, can also be improved by chelatable peptides or amino acids. Protein hydrolysates have a promising future in the mineral supplement market.

## Figures and Tables

**Table 1:** Iron-chelatable amino acids, peptides and proteins.

Class	Iron-Chelatable Substance	Sequence	Iron Valence	Reference
Amino acids	Arginine	R	II	[9]
	Aspartic acid	D	II	[10]
	Cysteine	C	III	[11]
	Glycine	G	III/II	[12,13]
	Glutamic acid	E	II/III	[14,15]
	Glutamine	Q	III	[16]
	Histidine	H	II/III	[9,11]
	Lysine	K	III	[11]
	Methionine	M	III	[16]
	Serine	S	III	[15]
	Threonine	T	II	[17]
Small peptides	Aspartame		II	[10]
	Arg-Glu-Glu	REE	II	[18]
	Asn-Cys-Ser	NCS	II	[19]
	Carbamyl glycine		II	[20]
	His-Tyr-Asp	HYD	II	[21]
	Isoleucyl-tryptophan	IW	II	[22]
	Leu-Ala-Asn	LAN	II	[19]
	Reduced glutathione	GSH	II	[19]
	Ser-Met	SM	II	[19]
	Ser-Cys-His	SCH	II	[23]
	Ser-Ala-Cys	SAC	II	[24]
	Val-Pro-Leu	VPL	II	[25]
Poly-peptides	α-lactalbumin and β-lactoglobulin hydrolysate		II	[25]
	β-casein peptide	PGPIPN	III	[26]
	Anchovy peptide	S(G)_7_LGS(G)_2_SIR	II	[27]
	Barley protein hydrolysate	SVNVPLY	II	[25]
	Buffalo α_S_-casein		II	[28]
	Caseinophosphopeptide	(SpSpSpEE)n	II	[29,30]
	Chickpea protein hydrolysate		II/III	[31]
	Cod skin peptides		II	[32]
	Ferrichrysin/ferrocins		III	[33]
	Hairtail protein hydrolysate		II	[21,34,35]
	Hydrolysate of Alaskan pollock skin	GPAGPHGPPG/SGSTGH	II	[23,24]
	Mackerel hydrolysate	NPVRGN/NPDRGN	II	[36,37]
	Lactein		II	[38]
	Peptide-hydroxamate	NAPVSIPQ	II/III	[39]
	Plasma hydrolysate	DLGEQYFKG	II	[40]
	Rice protein hydrolysate		II	[41]
	Scad protein hydrolysate		III	[42]
	Seaweed protein hydrolysate		II	[43]
	Sericin hydrolysate		II	[44]
	Shrimp protein hydrolysates	LPTGPKS	II	[45,46]
	Spirulina protein hydrolysate	TDPI(L)AACI(L)	II	[47]
	Soybean protein hydrolysate	DEGEQPRPFPFP	III/II	[48,49,50,51]
	Whey peptide		II	[52]
Protein	Ferritin		III	[53]
	Hen egg white lysozyme		III	[54]
	Thiolated human-like collagen		II	[55]
	Whey proteins		II	[56]

**Table 2:** The use of peptides or amino acids as iron supplements.

Class	Substance	Product Branch	Nation	References
Amino acids	The full spectrum of amino acids	FerrActiv^®^	America	[86]
	Glycine	Ferbisol^®^	Spain	[87]
Small peptides	Carbamyl glycine		China	[88]
Polypeptides	Deferrichrysin		Worldwide	[89]
	Donkey-hide gelatine	Dong E^®^	China	[90]
